# A Novel Screw Drive for Allogenic Headless Position Screws for Use in Osteosynthesis—A Finite-Element Analysis

**DOI:** 10.3390/bioengineering8100136

**Published:** 2021-10-01

**Authors:** Sebastian Lifka, Werner Baumgartner

**Affiliations:** Institute of Biomedical Mechatronics, Johannes Kepler University Linz, 4040 Linz, Austria; werner.baumgartner@jku.at

**Keywords:** allogenic bone screw, screw drive, headless bone screw, osteosynthesis, Finite-Element method (FEM), bioengineering

## Abstract

Due to their osteoconductive properties, allogenic bone screws made of human cortical bone have advantages regarding rehabilitation compared to other materials such as stainless steel or titanium. Since conventional screw drives like hexagonal or hexalobular drives are difficult to manufacture in headless allogenic screws, an easy-to-manufacture screw drive is needed. In this paper, we present a simple drive for headless allogenic bone screws that allows the screw to be fully inserted. Since the screw drive is completely internal, no threads are removed. In order to prove the mechanical strength, we performed simulations of the new drive using the Finite-Element method (FEM), validated the simulations with a prototype screw, tested the novel screw drive experimentally and compared the simulations with conventional drives. The validation with the prototype showed that our simulations provided valid results. Furthermore, the simulations of the new screw drive showed good performance in terms of mechanical strength in allogenic screws compared to conventional screw drives. The presented screw drive is simple and easy to manufacture and is therefore suitable for headless allogenic bone screws where conventional drives are difficult to manufacture.

## 1. Introduction

Especially in the case of complicated bone fractures, a simple plaster cast is often not enough to ensure good stiffening of the bone fragments. In such cases, a procedure called osteosynthesis, which serves to fix the individual fracture fragments to each other again in their original position and to re-stabilize the bone that has become unstable due to the fracture is used [1,2]. Typical areas of application are the stabilization of bones after fractures, stiffening operations on joints (arthrodesis) or on the spine (spondylodesis) and after osteotomy, a surgical technique in which bones are specifically cut to correct misalignments, e.g., the correction of hallux valgus [3,4]. The aim of osteosynthesis is therefore a stable fixation of the bone fragments to ensure a good healing of the fracture, to avoid of misalignments and to allow a partial or complete loading of the treated bones for an early functional post-treatment.

Osteosynthesis is done by surgical implementation of implants. Nowadays, a large number of various implants and techniques are available [5,6,7,8,9]. The focus in this paper is the screw osteosynthesis. Table 1 gives a short overview of the remaining types of osteosynthesis. In screw osteosynthesis, the bone fragments are fixed with screws [10,11] and are mostly based on the lag screw principal [12]. Here, the screw thread only engages the fracture part far from the head, while the screw slides freely in the bone near the head. This anchorage of the screw leads to the desired compression of the fragments [13,14]. This technique is often used for fractures close to a joint and for osteotomies in the forefoot. Today, many different screws are available, e.g., the Herbert screw, which creates the necessary compression by using two different thread pitches [15,16,17]. Besides lag screws, position screws are also used [18,19]. With position screws, a thread is cut in all fragments and thus the fragments are held in position without compression.

These screws are typically made of stainless steel [20], titanium [21,22,23] or biodegradable/bioabsorbable synthetic materials [24,25,26,27,28,29]. The advantages of screws made of bioabsorbable material include no implant removal required, no interference with MRI and no interference with future revision surgery if required. A major disadvantage of biodegradable screws is failure during insertion due to less stability compared to metal screws, e.g., in anterior cruciate ligament (ACL) reconstruction [30,31]. Furthermore, in some cases a foreign body reaction may occur [32].

**Table 1:** Overview of the remaining types of osteosynthesis.

Types of Osteosynthesis	Description
Kirschner-wire osteosynthesis	After reposition, the bone fragments are fixed with K-wires [33]. This type of osteosynthesis is suitable for fractures of small bones [34,35].
Tension-belt osteosynthesis	The principal of the tension-belt osteosynthesis is based on the fundamentals of reinforced concrete construction. In osteosynthesis, the compressive-resistant bone is combined with a tension-resistant wire [36,37,38].
Intramedullary nail osteosynthesis	In this method, a nail is placed intramedullary in the centre of the fractured bone and therefore it is a very load-table treatment for bone fractures [39,40,41,42].
Compound osteosynthesis	Due to osteoporosis, the fixation strength of implants is often drastically reduced. To increase stability, the principal of augmentation is applied [43]. In this process, areas with low bone density are filled with special bone cements to prevent the implant from breaking out [44,45].
External fixation	External fixation is primarily used for temporary stabilization of polytraumatized patients. In contrast to the other methods described, external fixation uses wires or screws that are attached to the bone fragments from the outside. [46,47,48].
Plate osteosynthesis	In plate osteosynthesis, a plate adapted to the size of the bone is placed over the exposed fracture and attached to all bone fragments with screws. This is how the plate provides stabilization of the bone [49,50,51].

Besides metal or biodegradable materials, such screws can also be made from cortical human donor bone [52]. These allogenic screws have very good osteoconductive properties and are therefore vascularized after 6 weeks and no longer visible on X-ray after 1 year [53,54]. These screws can be used as position screws for osteotomies, e.g., in hand, foot and knee surgery or as anchor screws for sutures and tendons and are available from 3.5 mm to 5 mm [53]. One can imagine that it is difficult to manufacture conventional screw drives like cross slot, hexalobular or internal hexagon in cortical bone screws as small micro cutters are required. Therefore, an easily manufacturable screw drive is needed for allogenic cortical bone screws. Common drives in allogenic bone screws are, on the one hand, an external hexagon, which is robust and easy to manufacture, but does not allow the screw to be screwed in completely. The other is a type of claw clutch, which is also easy to manufacture and allows a the screw to be screwed in completely, but the threads are interrupted in the area of the driver [53].

In this paper we introduce a novel, easy-to-manufacture screw drive for allogenic headless bone screws made of cortical human bone, which is fully internal and thus allows the screw to be fully inserted and has a thread along its entire length. In order to evaluate the strength of this drive, we performed simulations using the Finite-Element method (FEM) [55,56,57,58,59]. Furthermore, we validated the simulation using a screw made of human cortical donor bone produced by Surgebright (Neulichtenberg, Austria) [53]. Finally, we tested the novel screw drive experimentally on custom-made prototypes and compared the simulation results of the novel drive with simulation results of conventional drives. The validation of the simulation gave good results, so that the FEM-simulation allowed a fairly accurate prediction at which load and at which point the screw was likely to break. Compared to other conventional screw drives, the drive presented here has similar strength and is easy to manufacture.

## 2. Materials and Methods

### 2.1. Design of the Screw Drive and Minimal Screw Diameter

**Figure 1:**
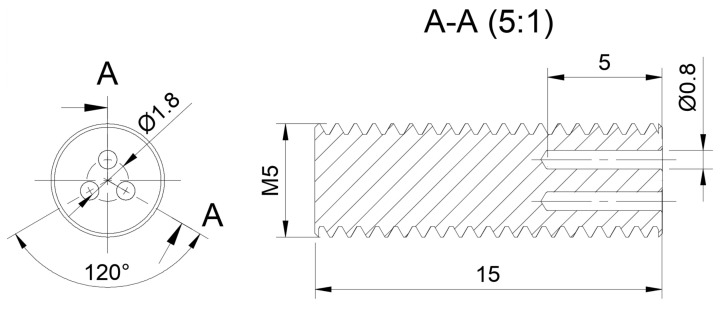
Basic design of a 5 mm-screw with the 3-bore drive. Units are in mm.

Since the primary goal of the screw drive for allogenic bone screws is a simple manufacturing process, the drive consists of only three bores arranged at an angle of 120°. Since two bores are unstable against tilting due to the additional degree of freedom and four bores take away additional material inside the screw, three bores were considered optimal. In the following, this drive will simply be referred to as the “3-bore drive”. Consequently, the insertion tool consists of three pins that are inserted into the bores of the screw. The bore diameter should be as small as possible to remove as little material as possible. If the hole diameter is too small, the insertion tool starts to bend, causing localized high stress peaks on the screw, which can lead to breakage. If the hole diameter is too large, too much material is removed from the inside of the screw, which also reduces stability. Through trial and error, a diameter of 0.8 mm turned out to be a good compromise. In addition, the insertion tool for the experiments was made using hardened pins, which are available with a minimum diameter of 0.8 mm. The depth of the bores was chosen to be 5 mm. This is a trade off between stability and reasonable manufacturability of the insertion tool. If the bores are chosen very deep, e.g., over the whole length of the screw, the pins of the insertion tool have to be very long, which makes them difficult to manufacture and very sensitive to bending. In addition, the highest stresses occur at the bottom of the pins, so deeper bores do not lead to increased stability (Figure 6). If, on the other hand, the bores are too short, the drive will be more sensitive to tilting. To ensure stability, we found that a minimum distance of about 0.7 mm should be maintained between the holes and the outer circumference of the screw. Therefore, the diameter of the round arrangement of the bores was chosen to be 1.8 mm. In order to maintain the 0.7 mm distance from the bore to the outer circumference of the screw, the core diameter must be at least 4 mm. This results in a minimum screw size of 5 mm with a standard M5 metric thread, which is common for allogenic bone screws [53]. So, the pitch circle diameter of 1.8 mm is a compromise between sufficiently strong connections between the holes and sufficient distance between the hole and the outer diameter to fit in a common 5 mm screw. To ensure complete screw-in, the outer diameter of the insertion tool must be smaller than the core diameter of the external thread. The technical drawing of the screw is shown in Figure 1.

### 2.2. CAD-Design

**Figure 2:**
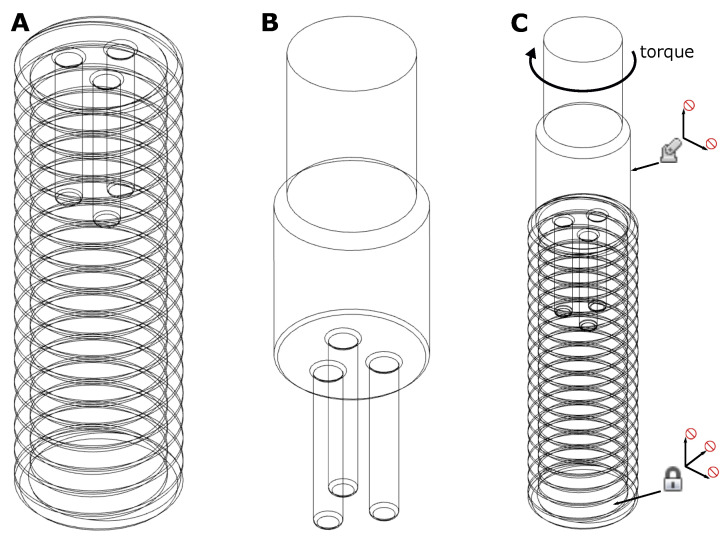
(**A**) CAD-model of a 5 mm M5 screw with the 3-bore drive; (**B**) CAD-model of the 3-bore drive insertion tool; (**C**) CAD-model of screw and insertion tool merged, which is the basis for the FEM-analysis.

3D CAD modelling and FEM-analysis were performed with Autodesk Fusion 360 (v.2.0.9930; Autodesk, Inc; San Rafael, CA, USA). To avoid singularities due to sharp edges [60] all edges were given a radius of 0.2 mm. The thread of the screw was fully modeled to simulate the behavior of the screw as well as possible (Figure 2A). The screw and the insertion tool were modeled separately, i.e., each as a separate file (Figure 2B). For the FEM-simulation, the two files were then merged, which led to a contact problem (Figure 2C). All screw drives were modeled form-fit and as simple as possible to achieve good simulation results. The underlying CAD-files can be found in [61].

### 2.3. Setup of the FEM-Analysis

The FEM-study was designed as a static stress analysis with linear isotropic material properties to reduce complexity and processing power. Since the idea is to find the torque that the screw can just withstand, i.e., the yield strength should not be reached, we remain in the linear range of the stress-strain diagram. This approach is therefore a good approximation [62]. For loads that exceed this maximum torque, the quality of this approximation deteriorates increasingly, but since it can be assumed that the screw will break at these loads, this fact is irrelevant for this application. The materials chosen for the simulation were human cortical bone for the screw and stainless martensitic chromium steel 1.4034 for the insertion tool, which is commonly used for surgical instruments. The material properties where chosen according to [53,62,63] for human cortical bone and chromium steel 1.4034 respectively (Table 2).

Structural constraints were applied on both the screw and the insertion tool, Figure 2C attempts to illustrate this graphically. The face of the screw opposite to the screw drive was fixed in all directions. The cylindrical face of the insertion tool was pinned in the radial and axial directions to prevent unwanted movement except for tangential movement due to the applied torque. Finally, a negative torque in z-direction was applied to the insertion tool as structural load. The contacts between the screw and the tool were defined using the “automatic contacts” function with the default contact tolerance of 0.10 mm. The average element size of the mesh was chosen between 1% and 10% based on the model and scaled per component. No adaptive meshing was used to increase computational speed. Each trial was solved using the “Cloud Solve” function of Fusion 360.

### 2.4. Evaluation of the FEM-Analysis

The evaluation of the results of the FEM-analysis was carried out by considering the von Mises stress distribution [57] at certain loads. Therefore, different loads/torques were applied as described in Section 2.3 and the resulting stress distribution was investigated. At the contacts between the insertion tool and the screw singularities and therefore areas with stresses much higher than the yield strength of cortical bone occur. If these high stress areas are only locally, i.e., the the absolute stress values drop rapidly with increasing distance from the contact point, it is very likely that the screw will withstand the load anyway. If, on the other hand, the high von Mises stresses are distributed over a large area, the screw is likely to fail at this load and above. So, if one knows the von Mises stress distribution at the respective applied load, with a little experience one can draw a conclusion as to whether the screw withstands the load or not. The aim is therefore to find the torque in the simulation that the screw can just withstand with regard to the von Mises stress distribution, i.e., the high stresses should only occur locally. At higher torques, it can then be assumed that the screw is likely to break. The peak values of the von Mises stresses were not taken into account because they are unrealistic high due to singularities, predominantly in the areas of the contact points between screw and insertion tool and at edges with small radius. All results of the FEM-simulations can be downloaded via the links provided in [61].

### 2.5. Validation of the FEM-Analysis

**Figure 3:**
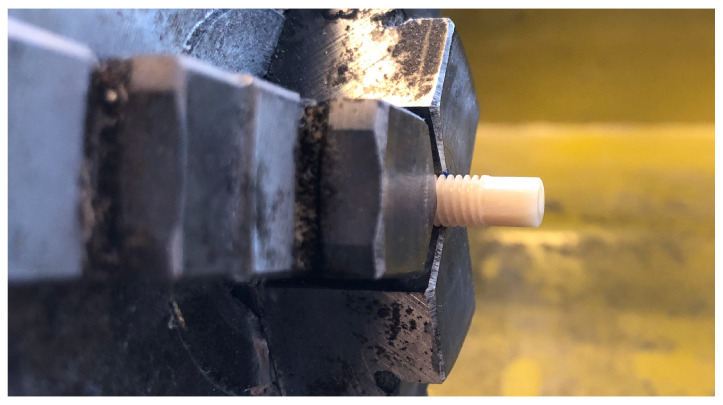
5 mm M5 prototype screw with external hexagon head made of cortical human donor bone for validation of the FEM-analysis fixed in a three-jaw chuck.

In order to verify that the performed simulations were correct and well represented the real behavior, the simulation result of a validation prototype was compared to a real prototype screw (Figure 3). The prototype was a 5 mm M5 screw with a 4 mm external hexagon head and a length of 30 mm made of human cortical donor bone. The external hexagon was chosen because it is a conventional head in allogenic screws [53] and insertion tools already exist. To test the strength of the real prototype, the screw was clamped in a three-jaw chuck and than loaded with a torque until material failure. The maximum torque was measured with an electronic torque adapter (Wisretec BDA2-030; Wisret Precision Co., Ltd; Shenzen, Guangdong, China). The prototype screw and the insertion tool were remodelled in Fusion 360 and subjected to FEM-analysis. The von Mises stress distribution was then studied and compared with the measurements of the real prototype in terms of failure torque and failure location.

### 2.6. Experimental Verification of the 3-Bore Drive

**Figure 4:**
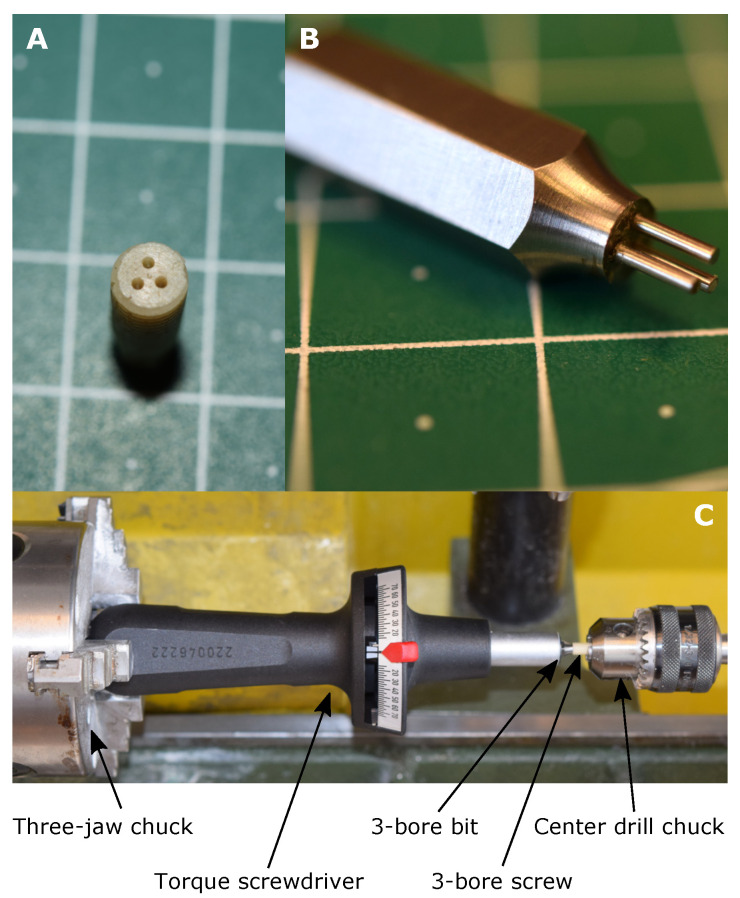
(**A**) Prototype screw of the 3-bore drive made of human cortical bone; (**B**) 3-bore drive insertion tool; (**C**) Failure torque measurement setup.

To verify the simulation results of the 3-bore drive, an experimental verification of the failure torque was carried out with a prototype screw, which is depicted in Figure 4A. For the prototypes, the same screws made from human cortical bone were used as for the validation, they were face turned and the three bores were drilled using a CNC-machine (PRO-BASIC-H 06/05, CNC-Modellbau, Gerabronn, Germany) and a 0.8 mm carbide drill (Bungard Elektronik GmbH & Co. KG, Windeck, Germany). The G-code was created with Fusion 360 and is available as extended data in [61]. The spindle speed was set to 10,000 rpm and the feed rate to 0.5 mm/s. The failure torque was measured with a torque screwdriver (Torsiometer Nr. 760/7,5, STAHLWILLE Eduard Wille GmbH & Co. KG, Wuppertal, Germany). Since the tool holder of the torque screw driver is designed for 1/4″-hexagon bits, the insertion tool for the prototype screw was manufactured as a hexagon bit by drilling three bores and inserting hardened 0.8 mm-pins into an already existing hexagon bit made of tool steel (Figure 4B). It must be mentioned that with this insertion tool a complete insertion of the screw is not possible, as this is not necessary for the verification of the failure torque. In Figure 4C the setup of the failure torque measurement is depicted. In order to minimize the bending moment on the screw during the application of the torque, the whole arrangement was mounted on a lathe as follows: The handle of the torque screwdriver was clamped into the three-jaw chuck, the 3-bore insertion tool was inserted into the screwdriver and the prototype screw was secured with the center drill chuck. The torque was then applied by hand on the three-jaw chuck until failure of the prototype screw.

### 2.7. Comparison with Conventional Screw Drives

To get an idea of the load that conventional screw drives are subjected to compared to the 3-bore-drive, a 2.5 mm internal hex key drive, a hexalobular (Torx) T10 drive, a simple 2×0.8 mm slot drive, which would be also easy to manufacture, and a claw clutch drive (used by [53]) were simulated. All CAD-files used for the simulation, except of the claw clutch drive, are available as extended data in [61].

## 3. Results

### 3.1. Validation of the FEM-Analysis

**Figure 5:**
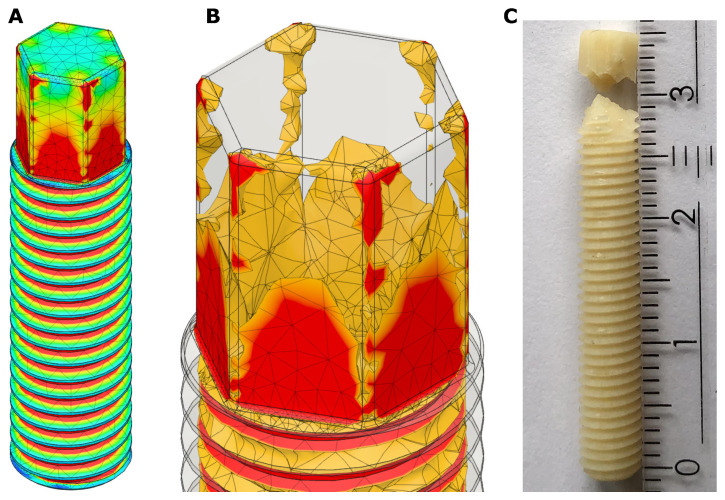
Validation of a 5 mm M5 prototype screw with external hexagon head made of cortical human donor bone. (**A**) Result of the FEM-analysis with a torque of 1250 Nmm (red zones represent areas with von Mises stress greater than 115 MPa); (**B**) Detail of the FEM-analysis with a torque of 1250 Nmm result, showing only the areas with von Mises stress greater than the yield strength of cortical bone (approx. 100 MPa [62]); (**C**) Picture of the real prototype after measurement of the failure torque.

Figure 5A,B shows the results of the FEM-analysis of the prototype screw, Figure 5C shows the real prototype after failure torque measurement. It can be seen that the real prototype failed at the bottom of the external hexagon head. The measurement of the failure torque was repeated three times with three different screws, the results are shown in Table 3. The mean failure torque was about 1250 Nmm. Therefore, the FEM-analysis was performed with a torque of 1250 Nmm (Figure 5A,B). The FEM-study shows the highest von Mises stress in the area of the bottom of the external hexagon, exactly where the real prototype also failed. The elements with von Mises stresses in the range and higher than the yield strength of the screw are widely distributed over the bottom of the hexagonal head, so it is very likely that the screw will fail at this point at the applied torque. The validation showed a good correlation between the FEM-simulation and the measurement of the failure torque of the real prototype.

### 3.2. FEM-Simulation of the 3-Bore-Drive

**Figure 6:**
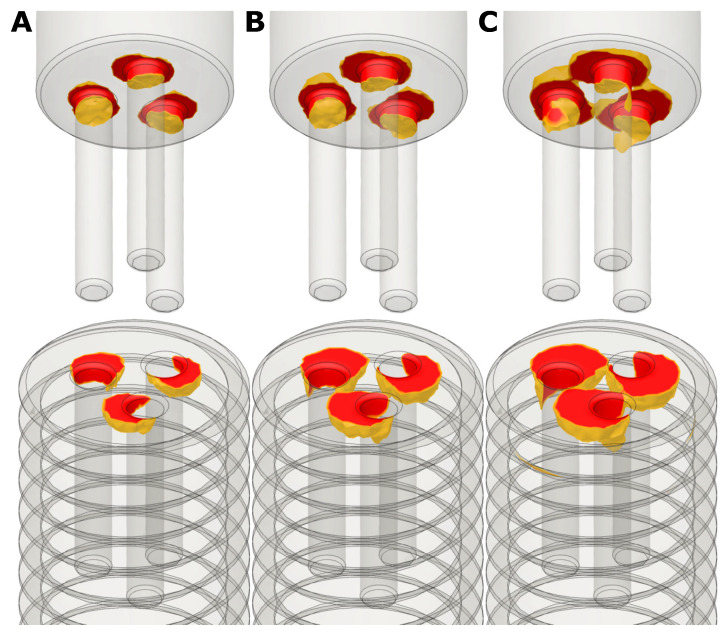
Results of the FEM-analysis of the 3-bore drive at different torques. Only the red zones with von Mises stresses higher than the yield stresses (100 MPa for the screw and 650 MPa for the insertion tool) are shown. (**A**) 200 Nmm; (**B**) 300 Nmm; (**C**) 400 Nmm.

Figure 6 shows the results of the FEM-analysis of the 3-bore drive. Figure 6A shows the von Mises stresses resulting from a torque of 200 Nmm applied to the insertion tool. In Figure 6B,C torques of 300 Nmm and 400 Nmm were applied. The red zones represent the elements with von Mises stresses grater than the yield strength of the screw (approx. 100 MPa) and the insertion tool (650 MPa). Elements with lower von Mises stresses are hidden for better visibility. One can see that at a torque of 200 Nmm (Figure 6A) the area with high von Mises stresses is only local, so it is very likely that screw and insertion tool will withstand the load. In contrast, at a torque of 300 Nmm and 400 Nmm (Figure 6B,C) the high von Mises stresses were distributed over a large area, even across the joint between the bores. So, it was very likely that the screw and the insertion tool failed under this load. The highest stresses for the insertion tool were at the bottom of the pins, so the total length of the pins did not matter for stability. One can assume that the 3-bore drive had a failure torque between approximately 200 Nmm and 300 Nmm.

### 3.3. Experimental Verification of the 3-Bore-Drive

**Figure 7:**
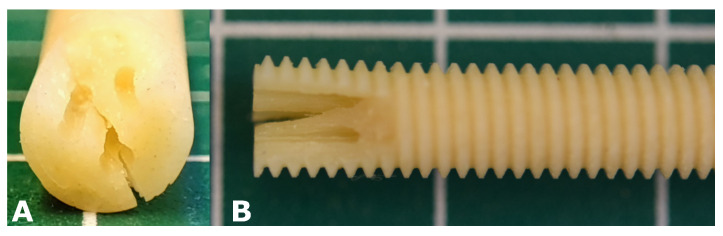
Prototype screw after failure torque measurement: (**A**) top view; (**B**) side view.

The results of the experimental verification of the 3-bore drive are listed in Table 4. The measurement was repeated five times, the mean value of the failure torque was 232.5 Nmm with a standard deviation of 24.37 Nmm. Figure 7 shows an example of a prototype screw after failure torque measurement, the point of failure in all measurements was the connection between the bores and resulting the connection between the bores and the outer circumference. The FEM-simulation predicted a failure torque between 200 Nmm and 300 Nmm at the connections between the bores, so the results matched very well, however the experimentally determined mean failure torque of 232.5 Nmm was in the lower range.

### 3.4. Comparison with Conventional Screw Drives

**Figure 8:**
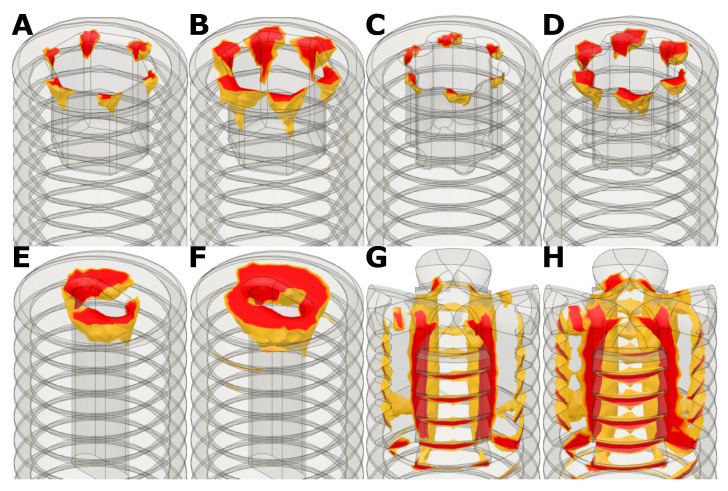
Results of the FEM-analysis of conventional drives at different torques. Only the red zones with von Mises stresses higher than the yield stresses (100 MPa for the screw) are shown. (**A**) 2.5 mm internal hexagon 200 Nmm; (**B**) 2.5 mm internal hexagon 300 Nmm; (**C**) Torx T10 200 Nmm; (**D**) Torx T10 300 Nmm; (**E**) 2 0.8 mm slot 200 Nmm; (**F**) 2 0.8 mm slit 300 Nmm; (**G**) Claw clutch 400 Nmm; (**H**) Claw clutch 500 Nmm.

In Figure 8 the simulation results of the conventional drives for comparison are depicted. The four drives were each simulated with two different torques. Figure 8A,B shows the internal hexagon drive with a torque of 200 Nmm and 300 Nmm. The highest stresses were located at the corners of the hexagon. Figure 8C,D shows the hexalobular Torx T10 drive again with 200 Nmm and 300 Nmm. Especially at the prongs of the Torx the stresses were very high. The simple slot drive is depicted in Figure 8E,F with 200 Nmm and 300 Nmm. One can see, that almost the entire area around the slot was subjected to very high von Mises stresses. Figure 8G,H shows the simulation results of the claw clutch drive at 400 Nmm and 500 Nmm. Here the highest stresses were located between the prongs of the drive where the insertion tool was placed.

Table 5 gives a summary overview of the results of all presented screw drives. One can see that the failure torque of the 3-bore-drive was approximately in the same range as the simulated failure torques of the internal hexagon and the hexalobular (Torx) drives, but with the advantage of being easier to manufacture. It is noticeable that the external hexagon drive had by far the highest failure torque. It can therefore be concluded that the external hexagon drive was basically the most stable one of all screw drives investigated, but had the disadvantage compared to the other drives that it did not allow the screw to be fully screwed in. The simple slot drive would also be easy to manufacture, but had a lower failure torque.

## 4. Discussion

In this paper we describe a simple screw drive for use in allogenic headless position screws made of human cortical bone. The screw drive is completely internal, i.e., no threads are removed, and it allows the screw to be fully inserted. Since it consists of only simple bores, this drive is easy to manufacture, which is crucial for allogenic screws.

In order to investigate the stability of the presented 3-bore drive, we performed simulations using the Finite-Element method. For simplicity, a linear, isotropic FEM was used. Since we want to find the torque that the screw can just withstand, i.e., the yield point should not be reached, we remain in the linear range of the stress-strain diagram. This approach is therefore a good approximation [62]. Nevertheless, a non-linear, orthotropic FEM-study would probably provide more accurate results and reduce singularities. However, the problem to be solved would be much more complex and therefore require more computing power. Furthermore, the simulation software used, Autodesk Fusion 360, does not support orthotropic materials in an FEM study.The comparison between simulation and experiment has shown that this approach is a sufficiently good approximation.

Since the problem investigated is a so-called contact problem, singularities occur at the contact points between screw and insertion tool and at edges with small radius. The stresses at these points are unrealistic high and therefore the maximum stress values were not considered. Here it is more important to see how far very high stresses extend over the component to get an impression of the stability. A validation of the simulation with a real prototype screw showed that the simulation reproduces the real behaviour well. One can conclude, that or simulation allows a fairly good estimation at which torque and at witch point the screw will fail.

In addition to the FEM simulations of the 3-bore drive, an experimental verification was carried out using custom-made prototype screws made from human cortical bone. The FEM-simulation predicted a failure torque between 200 Nmm and 300 Nmm, the experimentally determined failure torque is 232.5 Nmm, so it can be concluded that the results of the simulation and the experiment match very well. However, the mean experimental failure torque of 232.5 Nmm is in the lower range of the prediction of the simulation.

Comparisons of simulations with conventional screw drives showed, that the 3-bore drive has similar failure torques when used in allogenic cortical screws. The advantage of the 3-bore drive in allogenic screws is the simple manufacturing process. As one can imagine, it is difficult to manufacture a hex key drive or a hexalobular drive in cortical bone because the manufacturing process requires small and fragile micro cutters to achieve small radii e.g., for the hexalobular (Torx) drive. Therefore, the manufacturing process for conventional screw drives in cortical bone is difficult and time consuming.

The absolute value of the failure torque of the 3-bore drive is not very high at approximately 200 Nmm to 300 Nmm, since these screws are used as position screws, i.e., the screw is turned into a pre-cut thread, the occurring torques are rather low. Nevertheless, caution should be exercised when screwing in a screw with the suggested 3-bore drive.

We focused on screws with a diameter of 5 mm as this is the minimal diameter where this screw drive works properly. If the diameter gets smaller, there is not enough material inside the screw. A screw size of 5 mm is common for allogenic bone screws [53]. One can imagine that the 3-bore drive becomes more effective if the screw diameter is increased because there is more material inside the screw. The reasonable maximum screw size is about 8 mm, as the cortical portion of the raw bone is too thin for larger screw sizes. For an 8 mm screw with 1.5 mm bores, the simulated failure torque would be about 1200 Nmm. Therefore, the 3-bore drive represents a very stable, fully internal screw drive especially for larger, e.g., 8 mm, allogenic bone screws that can be used e.g., for fixation of a tendon graft where higher failure torques are required.

Furthermore, it must be emphasized that the proposed 3-bore drive is only intended for use in allogenic screws made of cortical bone. The 3-bore drive is not suitable for screws made of stainless steel, titanium or synthetic material, as conventional drives have better performance and are more stable.

Finally, Table 6 gives a brief overview over all presented screw drives regarding torque, manufacturing and the possibility of full screw-in. Compared to the external hexagon or the claw clutch, the 3-bore drive has a lower failure torque, but the external hexagon does not allow the screw to be fully screwed in. The claw clutch allows the screw to be fully screwed in, but part of the thread is removed, which is not the case with the 3-bore drive. The internal hexagon and the hexalobular drive have a similar failure torque, but are very difficult to produce in a cortical bone screw. The slot drive allows for complete screw-in and is also easy to manufacture, but the failure torque is lower compared to the other drives.

## 5. Conclusions

With the 3-bore drive a novel screw drive for allogenic headless position screws was introduced. The 3-bore drive is easy to manufacture which is crucial in bone screws made of human cortical donor bone. Furthermore, the 3-bore drive allows the screw to be fully inserted without removing any thread. Compared to conventional drives which allow a full screw-in, the 3-bore drive has a similar failure torque when used in cortical bone screws, but it is much easier to manufacture.

## Figures and Tables

**Table 2:** Mechanical material properties of the screw and the insertion tool used for the FEM-simulation.

	Human Cortical Bone	Chromium Steel 1.4034
Density in g/cm^3^	1.022	7.7
Young’s modulus in GPa	5	215
Poisson’s ratio	0.36	0.3
Yield strength in MPa	108	650

**Table 3:** Results of the failure torque measurement of the validation prototype screw.

n	Failure Torque in Nmm	Point of Failure
1	1200	bottom of hexagon head
2	1100	bottom of hexagon head
3	1400	bottom of hexagon head
Mean value	1233	-

**Table 4:** Results of the failure torque measurement of the 3-bore drive.

n	Failure Torque in Nmm	Point of Failure
1	200	connection between bores
2	250	connection between bores
3	262.5	connection between bores
4	225	connection between bores
5	225	connection between bores
Mean value	232.5	-
Standard deviation	24.37	-
Min. value	200	-
Max. value	262.5	-

**Table 5:** Overview of all presented screw drives.

Screw Drive	Failure Torque in Nmm (Simulated)	Failure Torque in Nmm (Experiment)
3-bore drive	200–300	230
External hexagon	1250	1233
Internal hexagon	200–300	-
Hexalobular (Torx)	200–300	-
Slot	100–200	-
Claw clutch	400–500	-

**Table 6:** Final evaluation of all presented screw drives regarding failure torque, manufacturing and the possibility of full screw-in.

Screw Drive	Torque	Manufacturing	Full Screw-In
3-bore drive	±	+	+
External hexagon	+	±	−
Internal hexagon	±	−	+
Hexalobular (Torx)	±	−	+
Slot	−	+	+
Claw clutch	+	±	±

## Data Availability

The data presented in this study are freely available in ”Extended data: A novel screw drive for allogenic headless position screws for use in osteosynthesis—A Finite-Element analysis“ at doi:10.5281/zenodo.4638924.

## Abbreviations

The following abbreviations are used in this manuscript:

FEM	Finite-Element Method
ACL	Anterior Cruciate Ligament
CAD	Computer-aided design
